# Phase I study of the humanised anti-EGFR monoclonal antibody matuzumab (EMD 72000) combined with gemcitabine in advanced pancreatic cancer

**DOI:** 10.1038/sj.bjc.6603083

**Published:** 2006-04-04

**Authors:** U Graeven, B Kremer, Th Südhoff, B Killing, F Rojo, D Weber, J Tillner, C ünal, W Schmiegel

**Affiliations:** 1 Department of Medicine, Ruhr University Bochum (Knappschaftskrankenhaus), In der Schornau 23-25, Bochum 44892, Germany; 2 Department of General Surgery and Thoracic Surgery, University Hospital of Schleswig-Holstein, Campus Kiel, Arnold-Heller Strasse 7, Kiel 24105, Germany; 3 Medical Oncology and Pathology, Vall d'Hebron University Hospital, P Vall d'Hebron 119-129, Barcelona 08035, Spain; 4 Departments of Clinical Sciences (Weber) and Clinicopharmacokinetics (Tillner), Merck KGaA, Frankfurter Strasse 250, F135/R222, Darmstadt D-64293, Germany; 5 Department of Biostatistics and Data Sciences, EMD Pharmaceuticals, 3211 Shannon Road, Durham, NC 27707, USA

**Keywords:** EMD 72000, epidermal growth factor receptor, gemcitabine, matuzumab, pancreatic cancer

## Abstract

The humanised anti-epidermal growth factor receptor (EGFR) monoclonal antibody matuzumab (formerly EMD 72000) is active against pancreatic cancer in preclinical studies. This phase I study assessed the safety and potential benefit of combined treatment with matuzumab and standard-dose gemcitabine. Three groups of chemotherapy-naive advanced pancreatic adenocarcinoma patients (*n*=17) received escalating doses of matuzumab (400 mg weekly, 800 mg biweekly, or 800 mg weekly) and gemcitabine (1000 mg m^–2^ weekly in weeks 1–3 of each 4-week cycle). Toxicity, antitumour activity, pharmacokinetic (PK) parameters, and pharmacodynamic (PD) markers in skin biopsies were evaluated. Severe treatment-related toxicities were limited to grade 3 neutropenia (*n*=3), leucopenia (*n*=1), and decreased white blood cell count (*n*=1). Common study drug-related adverse events were skin toxicities (grade 2=6, grade 1=7) and fever (grade 1=4). Matuzumab inhibited phosphorylated EGFR and affected receptor-dependent signalling and transduction; effects were seen even in the lowest-dose group. Pharmacokinetic data were consistent with results of matuzumab monotherapy. Partial response (PR) or stable disease occurred in eight of 12 evaluated patients (66.7%), with three PRs among six evaluated patients in the group receiving 800 mg weekly. Matuzumab in biologically effective doses with standard gemcitabine therapy appears well tolerated. The combination is feasible and may have enhanced activity.

The 5-year life expectancy of a patient diagnosed with pancreatic cancer is about 4% (Parkin *et al*, 1999). Gemcitabine therapy provides some benefit and modestly improves survival compared with fluorouracil, but median survival in patients with advanced disease remains less than 6 months (Burris *et al*, 1997). Altered expression or constitutive activation of the epidermal growth factor receptor (EGFR/HER1/erbB1) commonly occurs in both primary and metastatic pancreatic cancers and is often a critical component in progressive growth and resistance to normal mechanisms of cell death (Lemoine *et al*, 1992; Schmiegel *et al*, 1997; Xiong and Abbruzzese, 2002). Epidermal growth factor receptor expression in pancreatic cancer has been correlated with tumour aggressiveness (Tobita *et al*, 2003).

Matuzumab (formerly EMD 72000) is a humanised immunoglobulin G_1_ (IgG_1_) monoclonal antibody to the human EGFR (Kettleborough *et al*, 1991). Matuzumab binds the EGFR with high affinity, competitively blocking natural ligand binding and blocking receptor-mediated downstream signalling (Kettleborough *et al*, 1991; Tabernero *et al*, 2003). In preclinical studies, matuzumab demonstrated activity in the PAXF546 xenograft model of human pancreatic cancer that expressed high levels of EGFR and demonstrated almost complete resistance to clinically available chemotherapeutic drugs (Burger *et al*, 2003). Also, combining matuzumab with gemcitabine enhanced the effects of gemcitabine in the L3.6pl model of gemcitabine-sensitive pancreatic cancer (Amendt *et al*, 2003). In these studies, antitumour effects were mediated partly by direct inhibition of tumour growth and partly by inhibition of tumour-induced angiogenesis. In addition, because matuzumab is an IgG_1_ antibody to the EGFR able to mediate antibody-dependent cellular cytotoxicity, additional cytotoxic mechanisms may be involved in its effects in pancreatic cancer (Lo *et al*, 2003; Graeven *et al*, 2004).

A phase I clinical trial with matuzumab demonstrated that it is well tolerated as a single agent at doses between 400 and 1600 mg administered weekly, biweekly, or every 3 weeks (Vanhoefer *et al*, 2004). Dose-limiting toxicities (DLTs) occurred at 2000 mg on a weekly schedule and consisted of grade 3 headache and fever. Skin reactions, common with anti-EGFR agents (Thomas and Grandis, 2004), did occur with matuzumab, but results to date suggest that they are less severe than with other anti-EGFR agents and are restricted to grade 1 and 2 severity (Kollmannsberger *et al*, 2003; Salazar *et al*, 2004; Vanhoefer *et al*, 2004). Pharmacokinetic (PK) and pharmacodynamic (PD) results of the phase I study showed that matuzumab has predictable PKs at clinically relevant doses and that treatment with matuzumab blocks growth factor signalling through EGFR with a weekly dose of 800 mg as effectively as with doses of 1200 or 1600 mg.

This study was undertaken to determine whether matuzumab could be safely administered at biologically effective doses (800 mg weekly) with a standard regimen of gemcitabine to previously untreated patients with advanced pancreatic cancer and to study the PD and PK properties of matuzumab in this setting. In the absence of extensive prior clinical experience with matuzumab in combination with chemotherapeutic agents, the matuzumab dose was escalated through three treatment cohorts to the target weekly dose of 800 mg.

## PATIENTS AND METHODS

### Eligibility

Eligible patients had confirmed untreated stage III or IV pancreatic cancer, measurable by computed tomography (CT) or magnetic resonance imaging (MRI) and available tumour tissue for the determination of EGFR expression. Age ⩾18 years, Karnofsky performance status ⩾60%, life expectancy >12 weeks, and adequate organ and marrow function (white blood cell (WBC) count >3 × 10^9^ l^−1^, haemoglobin levels >9 g dl^−1^, platelet counts >100 × 10^9^ l^−1^, liver enzyme levels <2.5 × upper limit of normal (ULN), and creatinine levels <1.5 × ULN) were required. Pregnant or lactating females were ineligible, and females and males of childbearing age were required to use reliable birth control methods. Other exclusion criteria included treatment with any nonpermitted medication, the presence of brain metastases, active and uncontrolled infections, or uncontrolled severe organ dysfunction. All patients provided written informed consent. The ethical committees of the participating institutions approved the study. The study followed the Declaration of Helsinki and good clinical practice guidelines.

### Study design and treatment

The study (Protocol EMD 72000-021) was designed as a phase I, noncontrolled, nonrandomised, open-label study to evaluate the tolerability of matuzumab plus gemcitabine in patients with previously untreated advanced pancreatic cancer. Secondary objectives were to confirm previous PD findings about matuzumab-induced inhibition of EGFR-mediated cell signalling in the skin and potentially relate it to response, and to form a preliminary assessment of the activity of the matuzumab–gemcitabine combination in advanced pancreatic cancer. Other objectives were to evaluate matuzumab PKs and to confirm PK and PD results from previous studies. The study was conducted at two centres in Germany: the University-Hospital of Schleswig-Holstein in Kiel and Ruhr University in Bochum.

Matuzumab was supplied by Merck KGaA (Darmstadt, Germany). Matuzumab was administered to three patients in each dose group in an escalating, sequential manner in combination with a fixed dose of gemcitabine (1000 mg m^–2^ once weekly for 3 weeks, followed by a 1-week rest). Three matuzumab dose levels were planned on the basis of the prior studies (Vanhoefer *et al*, 2004): group I, 400 mg once weekly; group II, 800 mg every 2 weeks; and group III, 800 mg once weekly. If a DLT was observed in one of the three patients at one dose level, an additional three patients were enrolled at this dose level. If no DLT was observed at a given dose level, three patients were enrolled at the next dose level. If no DLT was observed in the first three patients at the highest dose (800 mg once weekly), three additional patients were enrolled and dose escalation was stopped. A maximum of 18 patients (six at each dose level) was planned. No intrapatient dose escalation was permitted.

During the first 8 weeks of study (two 4-week treatment cycles), data were collected to assess safety, PKs, and tumour responses. In a subset of patients who provided additional informed consent, PD studies were also conducted on skin biopsy specimens obtained before treatment and at the end of the first treatment cycle. After 8 weeks, treatment was discontinued in patients with progressive disease. Patients with stable or responding disease continued treatment until disease progression or unacceptable toxicity.

Matuzumab (Merck KgaA, Darmstadt, Germany) lyophilised in glass vials containing 200 mg antibody was reconstituted in 20 ml of sterile water, diluted with 0.9% (wt vol^−1^) normal saline solution to a total volume of 250 ml, and administered as a 1-h intravenous infusion. Patients were observed for 1 h after completion of the matuzumab infusion before being given the gemcitabine infusion. Commercially available gemcitabine was administered as a 30-min intravenous infusion.

### EGFR expression

Before entering the study, EGFR expression was established using a two-step immunohistochemical staining procedure on either archived tumour material or a recently obtained formalin-fixed, paraffin-embedded tumour biopsy. A commercial EGFR staining kit was used (Dako pharmDx, Dako Corporation, Glostrup, Denmark) and the manufacturer's recommended procedures were followed. This kit is approved by the US FDA as a type I *in vitro* diagnostic for use with routinely processed, paraffin-embedded specimens after fixation in 10% buffered neutral formalin. After proteolytic digestion, the samples are stained with a murine anti-human EGFR monoclonal antibody (Clone 2-18C9). The visualisation system is a secondary goat anti-mouse antibody and horseradish peroxidase. Results were interpreted by light microscopy. Tumours were considered positive if any membrane staining was observed in 10% or more of tumour cells.

### Evaluation of toxicities and response

Adverse events (AEs) were assessed weekly throughout the study, and toxicities were graded according to the National Cancer Institute Common Toxicity Criteria (NCI CTC; version 2.0). Dose-limiting toxicity was assessed only during cycles 1 and 2, and only possibly drug-related AEs were considered in defining the DLT. The maximum-tolerated dose (MTD) was defined as the dose level below that at which the DLT was observed in two or more of a maximum of six patients. Tumour response was assessed by CT or MRI of the target lesion(s) every 8 weeks and was classified as complete response (CR), partial response (PR), stable disease (SD) or progressive disease (PD) according to Response Evaluation Criteria in Solid Tumours (RECIST).

### Pharmacokinetics

Serum was collected at several time points to assess pharmacokinetic parameters (maximum serum concentration (*C*_max_), area under the serum concentration-versus-time curve (AUC), half-life (*t*_1/2_)) of matuzumab. For dose groups I and III (matuzumab 400 mg weekly or 800 mg weekly), 5 ml of venous blood was drawn before matuzumab administration, at 1, 2, 5, 48, and 96 h after the start of the first and fifth infusions and at 168 h thereafter (before the infusions on weeks 2 and 6). Predose samples were also collected before infusions 3, 4, 7, and 8. For dose group II (matuzumab 800 mg every 2 weeks), blood was drawn at the same time points. Serum concentrations of matuzumab were determined by Merck KGaA using a validated sandwich enzyme-linked immunosorbent assay with a lower limit of quantification (LLQ) of 0.5 *μ*g ml^−1^, as described previously (Vanhoefer *et al*, 2004).

The PK parameters of matuzumab were calculated according to noncompartmental methods using the PK software program Kinetica, version 4.1.1. The following parameters were determined from the serum concentration data of matuzumab: *C*_max_; time to reach *C*_max_ (*t*_max_); elimination *t*_1/2_; AUC up to time *t*, where *t* is the last time point at which a serum sample shows a concentration above the LLQ (AUC_0−*t*_); AUC until infinity (AUC_0−∞_), equivalent to AUC_0−*t*_+AUC_extra_, where AUC_extra_ represents an extrapolated value obtained by *C*_T_/*λ*_z_, where *C*_T_ is the last measured serum concentration above LLQ; volume of distribution during terminal phase (*V*_z_); and total-body clearance of drug from serum (CL).

### Pharmacodynamics

To assess the impact of matuzumab on signalling through the EGFR pathway, skin punch biopsy specimens were obtained from a subset of patients who provided additional informed consent. Biopsies were obtained before and after the first cycle (4 weeks) of treatment. Immunohistochemical analyses for pretreatment and on-treatment levels of basal EGFR (antibody=Dako, Clone 2-18C9, 1 : 1), phosphorylated EGFR (pEGFR) (Chemicon International Inc., Hampshire, England; Clone 174, 1 : 1000), phosphorylated p42/p44 mitogen-activated protein kinase (MAPK) at Thr202 and Tyr204 (pMAPK) (CST, #9101, 1 : 80), Ki-67 (Dako; MIB1, 1 : 100), p27^kip1^(Dako, Clone SX53G8, 1 : 100), and checkpoint kinase-1 (CK-1) were performed in paraffin-embedded sections, as described recently. Paired tissue sections known to be positive for the antigen were included in each analysis, and negative control sera were used on duplicate sections of the test materials (Albanell *et al*, 2001; Baselga *et al*, 2002). Slides were counterstained using Mayer's haematoxylin. The percentage of keratinocytes stained for each antibody was calculated based on counting the staining results in ≈1250 cells in interfollicular epidermis and in all the cells in the hair follicles (if present). Histopathologic scoring of stained cells was performed in a blinded manner. Pharmacodynamic studies were supervised by Dr F Rojo at the Vall d'Hebron University Hospital, Barcelona, Spain.

## RESULTS

This open-label nonrandomised dose-escalation study enrolled 17 patients with previously untreated advanced pancreatic adenocarcinoma; patient characteristics are shown in Table 1. Epidermal growth factor receptor expression was confirmed by immunohistochemistry in tumours from 16 patients; no tumour tissue was available for EGFR analysis for one patient.

**Figure 1:**
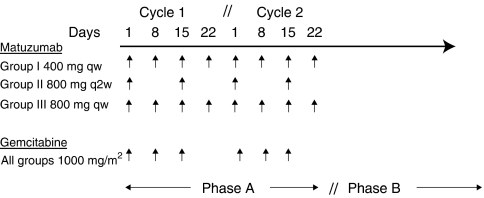
Dosing scheme for matuzumab and gemcitabine in the three groups in phases A and B.

The dosing schedule is shown in Figure 1. Among the 17 patients who entered the study, 12 completed at least two cycles (8 weeks) of treatment (phase A). Among patients not completing two cycles of treatment, four discontinued because of AEs and disease progression, and one owing to AEs alone. Nine patients received treatment after the second cycle (phase B) until disease progression (7) or death (2). The median duration of exposure to matuzumab was 99 days for the 17 patients in the treated population. A median of eight (1–45) infusions was administered.

Study drug-related AEs by NCI CTC grade and matuzumab dose are presented in Table 2. In the three treatment groups, one patient (400 mg matuzumab weekly) developed grade 3 neutropenia with a mild fever that did not meet the requirements for DLT (neutropenia with fever >40°C). Among the 17 patients in the study, there were five grade 3 haematologic AEs; none met the requirement for DLT. There were no other drug-related grade 3, or grade 4 AEs. Serious AEs occurred in 12 of 17 patients (70.6%), but none was drug related. Grade 3 nontreatment-related AEs occurring in more than one patient included fever (*n*=2), cholangitis (*n*=3), hypokalaemia (*n*=2), increased lactic dehydrogenase levels (*n*=2), and other increased liver enzyme levels (*n*=12). Grade 4 nontreatment-related AEs included cachexia, increased gamma-glutamyltransferase, and increased bilirubin in three of the five patients treated at 400 mg weekly with gemcitabine. Although it was not considered drug related by the investigator, a case of grade 4 Guillain–Barré syndrome with quadriplegia in a patient treated with matuzumab 800 mg weekly with gemcitabine was classified as possibly treatment related by the sponsor of the study. Three patients died owing to progressive disease while on treatment ⩽30 days since the last dose (one patient at each dose level). The most frequent drug-related AEs included grade 1/2 skin toxicity. Although the occurrence of skin toxicity was dose related, severity did not appear to be related to dose.

**Figure 2:**
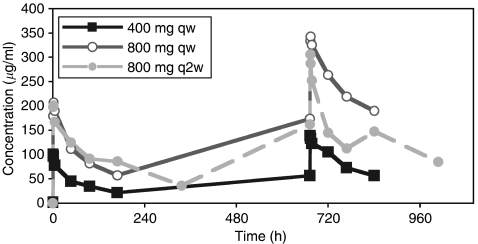
Mean serum concentration-versus-time curves of matuzumab. In all, 17 patients were assessable in week 1, and 12 patients were assessable in week 5.

Pharmacokinetic results are shown in Table 3, and serum concentration profiles in Figure 2, showing the relative differences among the dose groups. Serum samples were collected from all 17 patients but not at all time points. For all patients, a sufficient number of serum samples were available to allow calculation of PK parameters after first infusion (cycle 1). In cycle 2 (weeks 5–8), 12 patients (three at 400 mg weekly, three at 800 mg biweekly, six at 800 mg weekly) were included in the PK population, mainly because of discontinuation of patients. Peak serum concentrations were reached within 1–3 h after the start of the 1-h matuzumab infusion (Table 3). Exposure as indicated by the AUC and *C*_max_ showed nearly dose-proportional increases, with evidence of accumulation from weeks 1 to 5. Half-life tended to increase, and clearance appeared to decrease with matuzumab dose and the number of doses. Differences between the two 800-mg dose groups could be explained by the longer sampling interval for the biweekly-dose group. During week 5 at the 800-mg-weekly dose level, the mean half-life of matuzumab in four evaluable patients was about 8 days (196 h). The volume of distribution was about 5 l, consistent with the expected tissue distribution of a monoclonal antibody.

**Figure 3:**
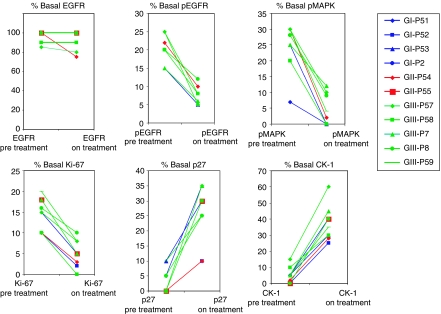
Percentage of basal keratinocytes expressing epidermal growth factor receptor (EGFR; *n*=10 patients), phosphorylated EGFR (pEGFR, *n*=9), phosphorylated p42/p44 mitogen-activated protein kinase (pMAPK) (*n*=8), Ki-67 (*n*=10), p27 (*n*=10), and CK-1 (*n*=10) determined by immunohistochemistry on pretreatment and week 4 skin biopsy specimens. Individual results are shown. Blue lines refer to patients who received matuzumab at 400 mg weekly, red lines 800 mg q 2 weeks, and green lines 800 mg weekly.

Results of PD studies on paired biopsy specimens of normal skin obtained from the same area of skin before treatment and after the first 4-week treatment cycle are shown in Figure 3. Separate permissions were sought to obtain the biopsy specimens, and 10 patients participated, with three in the 400-mg-weekly, two in the 80-mg-biweekly, and five in the 800-mg-weekly groups. For reasons related to the amount of tissue in each biopsy specimen, not all specimens could be tested for all markers. At all doses, matuzumab therapy inhibited signalling through EGFR (pEGFR) and the MAPK pathway, reduced the proportion of cycling cells in the biopsy specimen (Ki-67), and increased the expression of cell cycle inhibitory molecules (p27^kip1^, CK-1). Matuzumab did not affect the expression of EGFR, but its activation (pEGFR) was reduced in all specimens (mean 64.2%) after treatment. Activation of MAPK was reduced by a mean of 81.0% in eight paired biopsy specimens, and expression of Ki-67 in the basal keratinocytes was reduced by a mean of 65.3% in the 10 paired specimens. In 10 paired specimens, expression of the p27^kip1^ cyclin-dependent kinase inhibitor was increased from a mean basal level of 3–26.5% and that of CK-1 was increased from 4.7–37.3%.

Among the 12 patients evaluated for response after the second treatment cycle (8 weeks, phase A), PRs were seen in two of six patients (33%) in the group receiving 800 mg weekly, and six patients with SD were distributed across three dose groups, with two at 400 mg weekly, one at 800 mg biweekly, and three at 800 mg weekly. Best overall response after the second treatment cycle included the three PRs and five SDs, as in phase B, one patient in the group receiving 800 mg weekly with SD at the 8-week evaluation developed a sustained response. Median survival among the 17 patients was 3.7 months (range, 0.4–12.2 months).

## DISCUSSION

This phase I study showed that matuzumab at a biologically effective dose of 800 mg week^–1^ may be given safely with standard gemcitabine therapy to patients with advanced pancreatic cancer. Grade 3 treatment-related cases (total 5), including leucopenia (*n*=1), neutropenia (*n*=3), and decreased WBC count (*n*=1), occurred in the study, but their occurrence was unrelated to the matuzumab dose. There were 13 incidents at all dose levels of grade 1 or 2 skin toxicities. Adverse events in this study were consistent with those seen in other single-agent matuzumab studies (Vanhoefer *et al*, 2004). No DLTs were observed, which is also in agreement with previous work that established the MTD of single-agent matuzumab as 1600 mg on a weekly schedule (Vanhoefer *et al*, 2004).

Rash is the most common toxicity reported in patients treated with the anti-EGFR monoclonal antibodies cetuximab (Needle, 2002) and panitumumab (Schwartz *et al*, 2002), and in patients treated with the EGFR tyrosine kinase inhibitors gefitinib (Baselga *et al*, 2002) and erlotinib (Hidalgo *et al*, 2001), and its occurrence with these agents is occasionally severe. In this study, the severity of skin toxicity was limited to grades 1 and 2, with five of six grade 2 events observed with the lowest (400 mg weekly) dose and five of seven grade 1 events observed with the highest (800 mg weekly) dose. The pathophysiologic basis of skin rash in patients treated with EGFR signalling inhibitors is not clear.

Skin has been used as a surrogate for tumour in measuring the molecular effects of EGFR-targeted agents on EGFR, the ability of EGFR to transmit signals to kinases downstream in the signalling cascade and the responses mediated through EGFR, cell cycle progression and proliferation (Salazar *et al*, 2004; Tan *et al*, 2004). In this study, the molecular effects of matuzumab treatment on EGFR signalling were investigated in basal keratinocytes in skin biopsy specimens obtained before antibody treatment and on day 28 immediately before the fifth weekly dose. After the first cycle of matuzumab treatment, there was no effect on EGFR expression in basal keratinocytes, but EGFR signaling (pEGFR, pMAPK) was substantially reduced, the cellular growth fraction (Ki-67) was decreased to a similar extent, and accordingly, expression of cell cycle inhibitors was markedly increased. Tissue biopsies were obtained largely from patients receiving the highest matuzumab dose (800 mg weekly), but similar decreases in pEGFR, pMAPK, and Ki-67, and increases in p27^kip1^ and CK-1 were seen in biopsies from patients treated with lower doses. In a preceding study in which single-agent matuzumab (800, 1200, and 1600 mg) was administered on a weekly basis, highly significant changes in pEGFR, pMAPK, Ki-67, and p27^kip1^ expression were seen in skin biopsies obtained at day 28, but no quantitative differences were apparent among the three dose groups (Vanhoefer *et al*, 2004) with respect to any of these markers, and complete abrogation of pEGFR, pMAPK, and Ki-67 expression was not achieved even with the highest doses. The findings of the earlier study and the concordant results of the current study support the selection of 800 mg weekly as the optimal matuzumab dose for combination with standard gemcitabine therapy.

Pharmacokinetic data gave no indication that concurrent administration of matuzumab and gemcitabine affects the PKs of matuzumab. The PK values obtained are consistent with those obtained in matuzumab monotherapy studies (Tillner *et al*, 2003; Vanhoefer *et al*, 2004). As demonstrated in this and other studies, matuzumab PKs are predictable, although clearance is affected by the antibody dose, particularly at doses below 800 mg. A PK model for this dose effect proposes parallel elimination pathways for the antibody, one of which is saturable at the lower doses (Tillner *et al*, 2003). The half-life of matuzumab may be underestimated in this study, as the ratio of the sampling period to the calculated half-life was small, which could affect model-independent calculation of PK parameters. AUC_0−∞_ and half-life should be re-evaluated with an appropriately long sampling period.

Assessment of response was not a primary study objective, but it is worth noting that by the end of the second 4-week cycle, eight of the 12 evaluable patients benefited, with partial tumour responses in two of six patients (33%) in the group receiving 800 mg week^–1^ and stabilisation of the disease in eight other patients across the three dose groups. One patient with SD at the end of the second 4-week cycle had a partial tumour response after the third cycle. Encouraging findings have also been reported with the combination of gemcitabine and the anti-EGFR chimeric monoclonal antibody cetuximab in the first-line treatment of patients with advanced pancreatic cancer (Xiong *et al*, 2004). The study was similar to ours, except that the cetuximab study was a phase II design and the initial 7 weeks of gemcitabine were administered without rest. Among 41 patients, there were five PRs (12.2%) and 26 had SD. Median overall survival was 7.1 months with 31.7% 1-year survival, results somewhat better than those expected with gemcitabine alone (Burris *et al*, 1997).

This study shows that in patients with advanced pancreatic cancer, the combination of matuzumab and gemcitabine is well tolerated, with predictable PKs. In addition, weekly doses of matuzumab of 800 mg inhibited EGFR signalling and downstream effects associated with EGFR stimulation in skin biopsies. Responses in two of six patients treated with 800 mg weekly and disease stabilisation in an additional eight subjects across all dose groups suggest that a phase II evaluation of this regimen in a larger number of patients with advanced pancreatic cancer is warranted.

## Figures and Tables

**Table 1:** Patient characteristics

	**400 mg week^−1^ (*n*=5)**	**800 mg q2week^−1^ (*n*=4)**	**800 mg week^−1^ (*n*=8)**	**Total (*n*=17)**
Age – median in years (range)	64.0 (60–70)	55.5 (39–72)	69.5 (57–82)	64.0 (39–82)
Gender – male/female	4/1	1/3	4/4	9/8
Months from initial diagnosis to screening – mean (SD)	1.2 (0.84)	1.6 (0.71)	3.1 (3.19)	2.2 (2.35)
				
*Stage of tumour*				
III	1	1	1	3
IV	4	3	7	14
				
*Results of EGFR testing*				
Negative	0	0	0	0
Positive	4	4	8	16
Missing	1	0	0	1
Karnofsky performance status at screening – median % (range)	80 (60–100)	75 (70–80)	80 (70–90)	80.0 (60–100)

EGFR=epidermal growth factor receptor; SD=stable disease.

**Table 2:** Drug-related adverse events by NCI CTC grade

	**All (*n*=17)**
*Grade 3*	
Leucopenia	1
Neutropenia	3
WBC decreased	1
	
*Grade 2*	
Leucopenia	2
Thrombocytopenia	2
Nausea	2
WBC count decreased	1
Skin disorders	6
Hypotension	1
	
*Grade 1*	
Fever	4
Thrombocytopenia	1
Headache	1
Skin disorders	7
Folliculitis	1

DLT=dose-limiting toxicity; NCI CTC=National Cancer Institute Common Toxicity Criteria; WBC=white blood cell.

DLT was defined as grade 3 or 4 nonhaematologic toxicities (excluding headache, alopecia, nausea, vomiting, skin reactions, and fever above 40°C for less than 24 h); grade 4 nausea, vomiting, fever above 40°C sustained for more than 24 h; grade 3 or 4 neutropenia associated with complications (e.g. neutropenic fever); grade 4 thrombocytopenia, toxicity-related discontinuation of treatment for more than 1 week within the first two treatment cycles.

**Table 3:** Pharmacokinetic parameters of matuzumab in combination with gemcitabine

	***C*_max_ (*μ*g ml^−1^)**		***t*_max_ (h)**		**AUC_0−∞_ (h *μ*g ml^−1^)**		**AUC_0−*t*_ (h *μ*g ml^−1^)**		**AUC_extra_ (%)**		***t*_1/2_ (h)**		**CL (l h^−1^)**		***V*_z_ (l)**	
**Dose**	**Mean**	**SD**	**Mean**	**SD**	**Mean**	**SD**	**Mean**	**SD**	**Mean**	**SD**	**Mean**	**SD**	**Mean**	**SD**	**Mean**	**SD**
*400 mg week* ^−1^																
Week 1 (*n*=4–5)	101.2	20.8	1.6	0.9	10 228	1865	6988	1865	31.2	10.8	106.4	33.2	0.041	0.013	6.22	2.32
Week 5 (*n*=3)	138.0	7.0	1.0	0.0	24 698	3626	14 269	833	41.2	11.1	132.9	37.3	0.0281	0.0017	5.43	1.73
																
*800 mg q2week* ^−1^																
Week 1 (*n*=4)	208.5	44.2	2.0	0.8	37 657	15 289	26 730	8212	25.8	12.6	184.4	72.5	0.025	0.013	5.98	1.86
Week 5 (*n*=3)	303.3	21.5	2.0	1.7	78 704	344 238	45 695	12 328	37.6	15	260.2	105.7	0.0186	0.006	6.46	1.42
																
*800 mg week* ^−1^																
Week 1 (*n*=7–8)	212.1	41.2	2.1	1.3	27 854	9226	16 356	3433	38.7	11.4	134.6	43.7	0.0317	0.0108	5.69	1.35
Week 5 (*n*=4–6)	352.2	38.8	2.9	1.7	96 133	29 976	42 247	5961	54	10.4	196.0	55.2	0.0192	0.0029	5.36	1.28

AUC_0−∞_=area under the serum concentration-versus-time curve until infinity; AUC_0−*t*_=area under the serum concentration-versus-time curve up to time *t*, where *t* is the last time point at which a serum sample shows a concentration above the lower limit of quantification (LLQ); AUC_extra_=AUC from time *t* to infinity given as percentage from AUC_0−oo_; *t*_max_=time to reach *C*_max_; CL=total body clearance of drug from serum; *C*_max_=maximum serum concentration; SD=stable disease; *t*_1/2_=elimination half-life; *V*_z_=volume of distribution during terminal phase.
